# Physical Fitness Index and body mass index: A cross‐sectional study based on 1.3 million college students

**DOI:** 10.1371/journal.pone.0335194

**Published:** 2025-10-30

**Authors:** Sunchao Yin, Peng Jin, Qiang He, Yonghong Zhi

**Affiliations:** 1 College of Special Police, Nanjing Police University, Nanjing, China; 2 Department of Physical Education, Southeast University, Nanjing, China; 3 Department of Physical Education, Nanjing University of Finance and Economics, Nanjing, China; UC Los Angeles: University of California Los Angeles, UNITED STATES OF AMERICA

## Abstract

**Background:**

To explore differences in physical fitness levels among different grade groups and identify the association between body mass index (BMI) and physical fitness index (PFI).

**Methods:**

This study collected data from 1307857 participants (600999 females & 706858 males) who were undergraduate students aged from 18 to 24 years old. PFI was calculated using the z-scores of 5 sex-specific physical fitness items, namely sit-and-reach, broad jump, pull-up/sit-up, 50-meter dash, and 800/1000-meter run. BMI was classified into 4 categories based on the Asian standards recommended by the WHO: (1) BMI < 18.5 kg/m^2^; (2) 18.5 kg/m^2^ ≤ BMI < 23 kg/m^2^; (3) 23 kg/m^2^ ≤ BMI < 25 kg/m^2^; (4) BMI ≥ 25 kg/m^2^. ANOVA was used to detect variations in PFI among BMI categories and differences in physical fitness levels within grade groups. Finally, quadratic models were constructed to explore the association between BMI and PFI.

**Results:**

(1) An inverted “J” shape association was identified between BMI and PFI. (2) Both boys and girls in higher grades had higher PFI than those in lower grades.

**Conclusions:**

The association between BMI and PFI is non-linear. Physical development and physical activity engagement may assist in improving the physical fitness level of college students. Therefore, colleges should foster a more physical activity-friendly environment to reduce overweight and obesity rates, thereby enhancing fitness levels.

## Introduction

Obesity has emerged as a significant challenge globally: rates of obesity remain high for teenagers in wealthy regions and are steadily rising in low and middle-income countries [1]. Notably, the global prevalence of obesity is expected to exceed 18% in men and 21% in women, with severe obesity affecting 6% of men and 9% of women [2]. As one of the largest developing countries, over 50 percent of adults in China are considered overweight [3]. Calculated by the ratio of body weight to height, BMI (body mass index) is commonly used to evaluate obesity worldwide [4]. Reflecting the high overweight level, Chinese researchers observed the growth in BMI not only in urban, but also in rural areas [5]. The association was also discovered between high BMI and mortality rates [6]. Expect for its correlation with chronic diseases, a high BMI is also reported to be inversely correlated with fitness levels [7,8].

Fitness, a measure of an individual's capacity to perform efficiently and effectively in work and leisure activities, encompasses both physical fitness and cardiorespiratory fitness [9]. Physical fitness has recently garnered extensive attention owing to its correlation with various non-communicable diseases [10]. Noteworthily, a global decline in adolescents' aerobic performance from the late 1950s has been documented in earlier studies [11]. Long-distance running capabilities of Asian adolescents, including Chinese students, have significantly diminished [12]. Physical fitness is typically composed of muscle strength, muscle endurance, and motor ability [5]. While these fitness indicators vary globally, endurance running, explosive jump, dash, flexibility test, and strength test are generally included in the measurements [5,7,13]. Given the relatively high prevalence of physical inactivity in China, concerns have been raised regarding the decline in fitness levels [5]. Low physical fitness level is also recognized as a risk factor for multiple healthy problems. Numerous studies have identified relationships between low physical fitness levels and well-being. For instance, Dr Gunnar Erikssen reported even marginal enhancements in physical fitness were associated with a significant decrease in the risk of death [14]. Furthermore, correlations were noted between physical fitness and various chronic diseases, such as cancer and diabetes [15–17].

Over the past decade, a non-linear relationship has been established in the relationship between BMI and physical fitness index (PFI) [7,8,18]. Prior investigations have validated the quadratic relationship between BMI and PFI in Taiwanese youth [7]. A retrospective study conducted in 2019 also identified lower physical fitness levels in over-nourished and under-nourished youngsters compared to normal-weight youngsters [5].

Earlier studies reported differences in physical fitness across BMI categories by calculating P values [7,8,19]. However, significance can be exaggerated in large sample-size studies; thus, the effect size should be reported as an additional indicator. Therefore, this study uses effect sizes (Cohen's d) to assess the magnitude of differences. Moreover, earlier studies primarily concentrated on obesity and physical fitness levels in the pediatric population [5,7,19]. Besides the elementary and middle school years, collegiate years are also regarded as a crucial time for establishing lifelong healthy habits, such as maintaining physical activity and adopting nutritious dietary patterns [20–22]. Compared with the 1.5 million participants recruited in a study [5], the sample sizes in young adult investigations were relatively limited [8,18]. Chen's study primarily focused on freshmen within a single medical school, while Li's study sampled students from three universities located in different regions. In China, college students typically enter universities through the National College Entrance Examination and often migrate from various provinces to economically developed regions, such as Jiangsu, Beijing, or Shanghai. Consequently, there is a need to investigate whether the relationship between Body Mass Index (BMI) and Physical Fitness Index (PFI) observed in adolescents is consistent among college students. This requires survey encompassing participants from different grades and universities. Aim of this study is using a uniquely large dataset to examine variations in physical fitness levels across grade groups, and characterize the non-linear nature of the BMI-PFI relationship in young adults, a topic where previous studies had much smaller sample sizes.

## Method

### Participants

The data were extracted from a national fitness survey conducted among Chinese students in Jiangsu, China, in 2021. The authors accessed the identifiable participant data on September 5, 2024. Participants were informed through written consent. All measurements followed the Chinese National Student Physical Fitness Standard (CNSPFS) established in 2014 [23].

Participants in this study were undergraduate studentspursuing bachelor's degrees, representing colleges across Jiangsu Province. They were drawn from various regions across China, which enhances the sample's representatives of Chinese college students. All these tests were conducted in the physical education lessons or weekend, including enrolled undergraduate students. Those who were sick, disabled or do not wish to take the test were excluded to minimize confounding from pre-existing health conditions that affect both BMI and PFI.

While the initial sample size was 1394912 collected in 2021, researchers carried out a secondary sampling to exclude extreme values or data lacking important demographic information. Finally, 1,307,857 participants (600,999 females and 706858 males) aged from 18 to 24 years old were included in this research. The research was approved by IEC For Clinical Research of Zhongda Hospital, Affiliated to Southeast University (No. 2024ZDSYLL302-P01).

### Anthropometry measurement

#### Height.

During height measurement, students were instructed to stand barefoot with their backs against the measuring pillar. Additionally, they were instructed to maintain a naturally straight posture, with eyes looking forward, parallel to the ground, and a tightened torso. Arms were to be relaxed and hanging naturally on both sides of the body. Heels were to be placed together, with the heels, sacrum, and shoulder blades in contact with the measuring pillar.

#### Weight.

During weight measurement, students were required to remove heavy coats and items such as mobile phones and keys from their pockets. They were further instructed to stand barefoot at the center of the scale and maintain a stable posture.

Throughout the process, a staff member supervised the students to ensure that the correct posture was maintained and record the reading. BMI was calculated as weight (kg)/height (m)^2^ and expressed as kg/m^2^ [24].

### Physical fitness test

#### Sit-and-reach.

The sit-and-reach test was used to measure body flexibility. During the test, all students were instructed to remove their shoes and sit with their legs extended on the testing mat. At the start of the test, students were instructed to push the block forward with both hands as far as possible and release their hands at the maximum reach. One staff member supervised the students to ensure the correct posture was maintained, and another staff recorded the reading.

#### Broad jump.

The broad jump was employed to measure lower limb explosive power. Students were instructed to stand behind the starting line and jump forward as far as possible. The measurement was recorded from the heel of the furthest back foot after landing. During the test, a staff member supervised the students to ensure the proper procedure was followed, while another recorded distance.

#### Pull-up (for males)/ Sit-up (for females).

The pull-up and sit-up tests were utilized to measure muscle strength. For the pull-up test, students were instructed to grip the horizontal bar with palms facing forward before the test. The test involved pulling up until the chin exceeded the horizontal bar and then lowering until the angle between the upper arm and forearm exceeded 150 degrees, which counted as one repetition. Significant body swinging to gain momentum was prohibited. Regarding the sit-up test, students were instructed to lay flat on the mat with hands behind the head and legs bent at a 90-degree angle, with feet flat on the ground before the test. Each sit-up was counted when the elbows touched the knees, with the waist remaining in contact with the mat. During the test, a staff member supervised the students to ensure the correct procedure was followed and audibly counted the number of successful repetitions. Pull-ups/ sit-ups not performed according to the requirements were not counted.

#### 50-Meter Dash.

The 50-meter dash was used to assess speed and explosive power. Students were instructed to stand behind the starting line before the test and sprint to the finish line upon hearing the start command from the staff member. During the test, staff members were stationed at both the starting and finishing lines to monitor for no false starts and used a stopwatch to record the results.

#### 800/1000-Meter Run.

The 800-meter run for female students and the 1000-meter run for male students were used to evaluate cardiopulmonary and muscular endurance. Students were instructed to stand behind the starting line before the test began and run the full distance upon hearing the start command from the staff member. During the test, staff members were positioned along the track to supervise and ensure there were no false starts or course deviations and used a stopwatch to record the results.

The tests mentioned encompass multiple dimensions of physical fitness, including agility, explosive power, strength, and flexibility. The Physical Fitness Index (PFI), a widely applied metric for assessing physical fitness in adolescents and young adults, is used in this study to standardize fitness levels. PFI is calculated as the cumulative Z-score obtained from standardizing various physical fitness indicators within a student assessment system. The Z-scores were calculated separately for males and females but without stratification by grade level. To account for the fact that shorter times in the 50 m sprint, 1000 m run, and 800 m run signify better performance, the Z-scores for these endurance and sprinting events were inverted [25,26]; Therefore, PFI evaluates the overall physical fitness degree and is calculated by adding z-scores for sit-and-reach, broad jump, and pull-up/sit-up and subtracting the score for the distance run and dash.

### Statistics analysis

The BMI classification standards for Asians differ from those of other races. Therefore, this study adopted the BMI classification based on the Asian standards recommended by the WHO [27]. BMI was divided into four categories: BMI < 18.5 kg/m^2^; 18.5 kg/m^2^ ≤ BMI < 23 kg/m^2^; 23 kg/m^2^ ≤ BMI < 25 kg/m^2^; BMI ≥ 25 kg/m^2^. Descriptive statistics, including mean and standard deviations, were used to assess physical fitness levels across the BMI and grade groups. ANOVA was used to identify differences in PFI across the BMI and grade categories. Cohen's d was applied to compute effect sizes between each pair and classified as follows: 0.2, 0.5, and 0.8 for small, medium, and large effect sizes, respectively.

A quadratic regression model was applied to analyze the relationships between body mass and physical fitness in each grade group:


$$PFI={aBMI}^{\text{2}}+bBMI+c$$

where a, b, and c are constant. Statistical analyses were carried out using R version 4.4.1 (University of Auckland, Oakland, New Zealand).

## Results

While ANOVA identified significances across grades, effect sizes were negligible for most items. For females, significant differences were identified in the sit-and-reach and dash tests, indicating a decline from older to younger grades (Tables 1 and 2). For males, significant differences were noted in the sit-and-reach, dash, and pull-up tests. Similar to females, although sophomore and junior did not indicate significant differences in dash, performance in sit-and-reach and dash were also lower in lower grades for males (Tables 3 and 4). Comparatively, juniors outperformed others in the pull-up test. Across both sexes, PFI was higher among students in higher grades compared with those in lower grades (Tables 1–4).

**Table 1:** Comparisons within different grade groups (female).

Grade	N	Age	800-Meter Run (Seconds)	50-Meter Dash (Seconds)	Broad Jump (Centimeter)	Sit-and-reach (Centimeter)	Sit-up (Times)	BMI	PFI
1	172302	18.24 (0.60)	256.44 (31.05)	9.35 (0.80)	166.93 (18.31)	17.13 (6.97)	35.99 (8.41)	21.32 (3.49)	−0.31 (3.07)
2	178294	19.05 (0.67)	257.52 (31.79)	9.31 (0.81)	168.19 (18.83)	17.84 (6.80)	37.04 (8.51)	21.09 (3.37)	0.01 (3.10)
3	158309	20.08 (0.75)	260.69 (33.54)	9.30 (0.82)	168.66 (18.04)	18.04 (6.64)	37.76 (8.66)	20.89 (3.23)	0.08 (3.08)
4	92094	21.03 (0.82)	262.15 (35.02)	9.27 (0.77)	169.06 (17.37)	19.16 (6.74)	39.38 (8.68)	20.82 (2.94)	0.43 (2.94)
Total	600999	19.39 (1.20)	258.76 (32.63)	9.31 (0.80)	168.09 (18.27)	17.92 (6.83)	37.29 (8.62)	21.06 (3.31)	−0.00 (3.08)
Comparisons			1 > 2 > 3 > 4	4 > 3 > 2 > 1	4 > 3 > 2 > 1	4 > 3 > 2 > 1	4 > 3 > 2 > 1	1 > 2 > 3 > 4	4 > 3 > 2 > 1

Note: Data are mean (SD) in the main part, and category (unit) in the first row. BMI is indicated by raw scores. PFI is indicated by z-scores. ANOVA results indicated statistically significant differences among grade groups for all variables (P < 0.05). “>” and “<” indicate statistically significant results of post-hoc pairwise comparisons (P < 0.05). PFI is the contraction of Physical Fitness Index.

**Table 2:** Effect sizes for PFI comparisons within different grade groups (female).

Effect size	800-Meter Run	50-Meter Dash	Broad Jump	Sit-and-reach	Sit-up	BMI	PFI
**1/2**	−0.01 (negligible)	−0.12 (negligible)	−0.01 (negligible)	−0.09 (negligible)	−0.06 (negligible)	−0.04 (negligible)	−0.28 (small)
**1/3**	−0.02 (negligible)	−0.26 (small)	−0.02 (negligible)	−0.16 (negligible)	−0.10 (negligible)	−0.08 (negligible)	−0.49 (small)
**1/4**	−0.03 (negligible)	−0.39 (small)	−0.03 (negligible)	−0.27 (small)	−0.17 (negligible)	−0.13 (negligible)	−0.76 (medium)
**2/3**	−0.02 (negligible)	−0.14 (negligible)	−0.01 (negligible)	−0.07 (negligible)	−0.05 (negligible)	−0.04 (negligible)	−0.21 (small)
**2/4**	−0.03 (negligible)	−0.29 (small)	−0.02 (negligible)	−0.18 (negligible)	−0.12 (negligible)	−0.09 (negligible)	−0.48 (small)
**3/4**	−0.01 (negligible)	−0.16 (negligible)	−0.01 (negligible)	−0.11 (negligible)	−0.07 (negligible)	−0.05 (negligible)	−0.25 (small)

Note: Effect sizes were computed by Cohen’s d between each pair and classified as follows: 0.2, 0.5, and 0.8 for small, medium, and large effect sizes, respectively. BMI is the contraction of Body Mass Index. PFI is the contraction of Physical Fitness Index.

**Table 3:** Comparisons within different grade groups (male).

Grade	N	Age	1000-Meter Run (Seconds)	50-Meter Dash (Seconds)	Broad Jump (Centimeters)	Sit-and-reach (Centimeters)	Pull-up (Times)	BMI	PFI
**1**	211463	18.32 (0.74)	260.00 (38.60)	7.57 (0.68)	222.65 (23.04)	13.39 (7.82)	5.39 (5.11)	23.07 (4.20)	−0.12 (3.08)
**2**	208673	19.13 (0.72)	262.69 (40.04)	7.55 (0.72)	223.89 (23.25)	13.46 (7.75)	5.93 (5.40)	22.93 (4.12)	0.01 (3.16)
**3**	188148	20.14 (0.79)	266.13 (42.29)	7.56 (0.74)	224.15 (22.68)	13.92 (7.64)	6.38 (5.69)	22.83 (3.96)	0.06 (3.20)
**4**	98574	21.07 (0.95)	269.81 (44.88)	7.54 (0.66)	225.81 (22.09)	15.03 (8.10)	5.89 (5.58)	23.05 (3.71)	0.12 (3.14)
**Total**	706858	19.43 (1.23)	263.79 (41.07)	7.56 (0.70)	223.86 (22.90)	13.78 (7.81)	5.88 (5.43)	22.96 (4.05)	−0.00 (3.15)
**Comparisons**			1 > 2 > 3 > 4	4 > 2 ≈ 3 > 1	4 > 3 > 2 > 1	4 > 3 > 2 > 1	3 > 2 > 4 > 1	1 ≈ 4 > 2 > 3	4 > 3 > 2 > 1

Note: Data are mean (SD) in the main part, and category (unit) in the first row. BMI is indicated by raw scores. PFI is indicated by z-scores. ANOVA results indicated statistically significant differences among grade groups for all variables (P < 0.05). “>” and “<” indicate statistically significant results of post-hoc pairwise comparisons (P < 0.05), whereas “≈” indicates no statistically significant difference (P > 0.05). BMI is the contraction of Body Mass Index. PFI is the contraction of Physical Fitness Index.

**Table 4:** Effect sizes for comparisons within different grade groups (male).

Effect size	1000-Meter Run	50-Meter Dash	Broad Jump	Sit-and-reach	Pull-up	BMI	PFI
**1/2**	−0.01 (negligible)	−0.16 (small)	−0.01 (negligible)	−0.07 (negligible)	−0.18 (negligible)	−0.04 (negligible)	−0.24 (small)
**1/3**	−0.03 (negligible)	−0.34 (small)	−0.02 (negligible)	−0.16 (negligible)	−0.35 (small)	−0.08 (negligible)	−0.44 (small)
**1/4**	−0.05 (negligible)	−0.51 (medium)	−0.03 (negligible)	−0.28 (small)	−0.42 (medium)	−0.14 (negligible)	−0.65 (medium)
**2/3**	−0.02 (negligible)	−0.19 (negligible)	−0.01 (negligible)	−0.09 (negligible)	−0.17 (negligible)	−0.04 (negligible)	−0.20 (negligible)
**2/4**	−0.03 (negligible)	−0.39 (small)	−0.02 (negligible)	−0.22 (small)	−0.23 (small)	−0.10 (negligible)	−0.40 (small)
**3/4**	−0.02 (negligible)	−0.22 (small)	−0.01 (negligible)	−0.14 (negligible)	−0.06 (negligible)	−0.06 (negligible)	−0.19 (negligible)

Note: Effect sizes were computed by Cohen's d between each pair and classified as follows: 0.2, 0.5, and 0.8 for small, medium, and large effect sizes, respectively. BMI is the contraction of Body Mass Index. PFI is the contraction of Physical Fitness Index.

Concerning BMI classifications, groups 1 and 2 displayed the highest performance, whereas group 4 exhibited the poorest performance, irrespective of gender. Comparisons between groups 1 and 2 yielded negligible effect sizes in both males and females (Table 5). Similarly, comparisons between groups 1 and 3 also yielded negligible effect sizes in males.

**Table 5:** PFI Comparisons within different BMI groups.

	BMI Class	1	2	3	4	
**Female**	N	121713	350267	64261	64758	
	PFI	0.29 (3.05)	0.27 (3.03)	−0.50 (2.95)	−1.53 (3.09)	
	1/2	1/3	1/4	2/3	2/4	3/4
**Effect Size (Female)**	0.00 (negligible)	0.26 (small)	0.59 (medium)	0.26 (small)	0.60 (medium)	0.34 (small)
**Male**	N	75025	328884	119196	183753	
	PFI	0.53 (2.89)	0.79 (2.98)	−0.01 (2.91)	−1.63 (3.08)	
	1/2	1/3	1/4	2/3	2/4	3/4
**Effect Size (Male)**	0.09 (negligible)	0.19 (negligible)	0.71 (medium)	0.27 (small)	0.80 (large)	0.54 (medium)

Note: BMI is the contraction of Body Mass Index, and indicated by the raw score. PFI is the contraction of Physical Fitness Index, and indicated by the z-score. Data are mean (SD) in the main part. Effect sizes were computed by Cohen's d between each pair and classified as follows: 0.2, 0.5, and 0.8 for small, medium, and large effect sizes, respectively.

**Fig 1:**
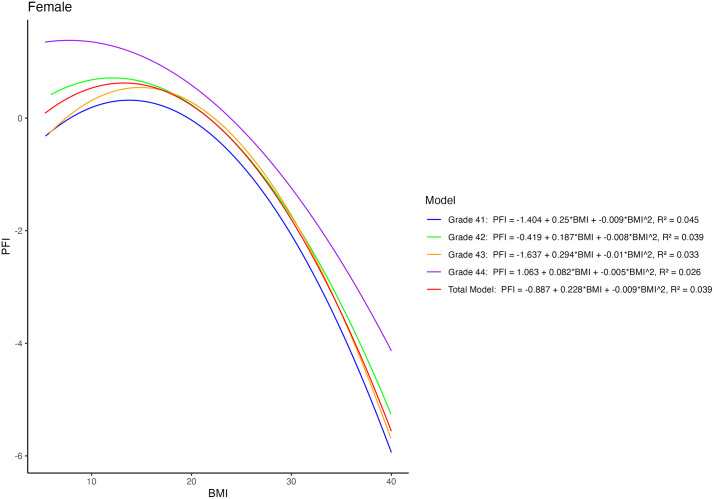
Quadratic relationship between PFI and BMI for females of different grades.

**Fig 2:**
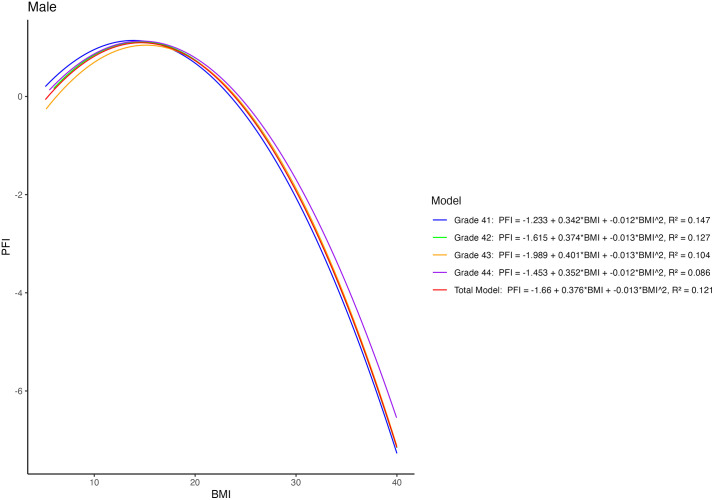
Quadratic relationship between PFI and BMI for males of different grades.

In accordance with the results of ANOVA, regression results also displayed lower PFI in participants with high BMI (Figs 1 and 2). As anticipated, regression models for each grade displayed a “J” shape. Regardless of gender, participants with high BMI levels generally had low physical fitness performance. For females, senior students had higher PFI compared to the other group-specific students at any BMI level. In contrast, female freshmen had the lowest PFI in most BMI categories. Meanwhile, the difference in PFI among different BMI categories was relatively lower in females compared with males. Despite the relatively minor differences, male seniors still showed a higher PFI within their grade group, especially among those with a high BMI.

## Discussion

The research results indicate that, regardless of gender, senior college students demonstrated significantly lower physical fitness compared to junior students. Similarly, students with higher BMI showed poorer performance than those with lower BMI, with significant differences observed in certain fitness tests. Physical fitness levels were compared across sex-specific grade and BMI groups. Significant differences and effects were observed in the dash and sit-and-reach tests among female groups.

As an assessment of muscle strength, the sprint test reflects explosive power and agility [28]. Notably, greater muscle strength was observed at higher grade levels among both males and females. A previous study reported differences in 50-meter dash performance among Chinese student cohorts, with relatively stronger outcomes observed even during the isolation period [29]. Although sprint performance predominantly relies on genetic features, some determinants, such as technique, power, and sprint-specific endurance, can be optimized by training [30].

In addition to improvements in dash performance, subjects in higher grade groups also demonstrated superior flexibility than those in the lower grade groups. As a validated measurement for hamstring flexibility, sit-and-reach tests are practical evaluations [31]. Recently, an increasing number of colleges have introduced yoga and dance into physical education curricula in China [32,33]. Compared with traditional strength training courses in middle school, yoga and dancing courses emphasize flexibility, thereby improving flexibility among college students [34].

Despite being in the last position, junior male students achieved optimal results in the pull-up test. This phenomenon can be ascribed to the curriculum of Chinese colleges, which typically do not include final examinations for physical education courses in the final two years. Hence, students do not have to undergo pull-up training to meet the standards [35]. In the past few decades, underperformance in pull-ups has remained a persistent issue among adolescents and young adults [36]. A study has reported a decline in pull-up performance between 2013−2017 in Chinese college students. Also, a reduction in maximum pull-up repetitions was noted during COVID-19 by researchers from Tsinghua University [29]. Consequently, enhancing students' involvement in extra-curricular physical activities may potentially address this issue [37].

Although variations were noted in specific items, senior students had better physical fitness performance than senior students, consistent with the results of the regression model across different grade groups. In contrast, longitudinal studies investigating physical fitness reported opposite findings; specifically, a decline in physical fitness levels was detected in teenagers aged 15–18 years [5]. Compared with young adults, the majority of teenagers aged 15–18 years are in senior high school and face high pressure due to high-school entry examinations [5]. In China, intense academic competition was suggested to be correlated with childhood obesity [38].

Improvements in physical fitness levels can also be attributed to physical development in adulthood. As individuals progress through puberty, those in their early twenties experience a peak in physiological development, especially in muscle strength, reaction time, sensory abilities, and cardiac function [39]. Furthermore, higher physical activity levels were found to be correlated with greater physical development, such as increased bone mineral density and neuromotor fitness growth [40]. Therefore, there is a pressing need for policymakers and colleges to develop various strategies to promote physical activity.

According to prior investigations, parabolic models can be used to describe the relationship between BMI and PFI in young adults [8,18]. A study enrolling 8548 college students presented a regression model with an inverted “J” shape, whereas another study displayed an inverted “U” shape in 2023 [8,18]. Variations between the two models can be ascribed to the physical fitness outcomes in low BMI categories. Although the confidence intervals are wide in at the extremes of the BMI range, the results of this study found an inverted “J” shape model, consistent with the study conducted in 2020 [8].

According to the regression model and ANOVA results within BMI groups, the difference between students with low BMI (group 1) and normal BMI (group 2) was not significant in both males and females. Nonetheless, students with high BMI (groups 3 and 4) significantly underperformed in physical fitness measurement. As is well documented, overweight is a chief determinant of low fitness performance [5,7,8,18,19]. According to BMI trend research published in the Lancet, the prevalence of obesity in China rose from mid-rank in 1975 to second place for both males and females in 2014 [2]. Evidently, this upward trend is associated with Chinas reforms and policies, which contributed to substantial economic growth during the last few decades. It is worthwhile acknowledging that obesity and limited physical fitness are impacted by numerous sources, such as overnutrition. Based on the China Health and Nutrition Survey (CHNS), the consumption of animal-sourced foods, Sugar-sweetened beverages, and oil has gradually increased since 1989 [41]. Furthermore, physical inactivity and sedentary behavior are conducive to weight gain and low physical fitness [42].

As recommended by the 2020 WHO guideline, adults ought to engage in a minimum of 150−300 minutes of moderate-intensity aerobic physical activity per week or at least 75−150 minutes of vigorous-intensity aerobic activity [43]. Pedro and his colleagues reported that approximately 31.5% of individuals aged 15 years or older were physically inactive, whilst around 40% to 50% of college students did not meet the activity level suggested by WHO [44,45]. Furthermore, the lockdown caused by COVID-19 exacerbated physical inactivity in China [46]. Besides physical education courses, colleges can enhance student engagement by offering various recreational facilities, organizing intramural sports and clubs, promoting active transportation, and hosting fitness events.

This study has some limitations that warrant acknowledgment. To begin, given the cross-sectional nature of the analysis, a causal relationship could not be established between BMI and PFI. Secondly, subjects were only recruited from colleges in Jiangsu province, thereby limiting the generalizability of our findings. As one of China's most economically developed provinces, Jiangsu attracts students from across the country; however, the sample remained unbalanced. Moreover, the relatively low R^2^ values observed in our quadratic regression models are not unexpected. Beyond BMI, economics status, lifestyle behaviors and environment can also be correlated to one's physical fitness level [47]. BMI is widely applied for its conveniences in measuring and calculating, but cannot distinguish between muscle and fat mass as an anthropometric index [48]. This should be interpreted as evidence of BMI being an independent but modest correlate, rather than a strong predictor, of physical fitness in students. Further comprehensive and prospective cohort studies are warranted to validate our results.

## Conclusion

Overall, being overweight negatively affects college students' physical fitness levels. However, physical development and increased engagement in physical activity during adulthood could potentially help mitigate these effects. It is crucial for colleges to implement targeted interventions and foster a more activity-friendly environment to reduce overweight prevalence and enhance overall fitness levels.
